# Comparative Genomics of Multidrug Resistance-Encoding IncA/C Plasmids from Commensal and Pathogenic *Escherichia coli* from Multiple Animal Sources

**DOI:** 10.1371/journal.pone.0023415

**Published:** 2011-08-12

**Authors:** Claudia Fernández-Alarcón, Randall S. Singer, Timothy J. Johnson

**Affiliations:** 1 Department of Veterinary and Biomedical Sciences, College of Veterinary Medicine, University of Minnesota, St. Paul, Minnesota, United States of America; 2 Instituto de Medicina Preventiva Veterinaria, Facultad de Ciencias Veterinarias, Universidad Austral de Chile, Valdivia, Chile; Universität Münster, Germany

## Abstract

Incompatibility group A/C (IncA/C) plasmids have received recent attention for their broad host range and ability to confer resistance to multiple antimicrobial agents. Due to the potential spread of multidrug resistance (MDR) phenotypes from foodborne pathogens to human pathogens, the dissemination of these plasmids represents a public health risk. In this study, four animal-source IncA/C plasmids isolated from *Escherichia coli* were sequenced and analyzed, including isolates from commercial dairy cows, pigs and turkeys in the U.S. and Chile. These plasmids were initially selected because they either contained the *floR* and *tetA* genes encoding for florfenicol and tetracycline resistance, respectively, and/or the *bla_CMY-2_* gene encoding for extended spectrum β-lactamase resistance. Overall, sequence analysis revealed that each of the four plasmids retained a remarkably stable and conserved backbone sequence, with differences observed primarily within their accessory regions, which presumably have evolved via horizontal gene transfer events involving multiple modules. Comparison of these plasmids with other available IncA/C plasmid sequences further defined the core and accessory elements of these plasmids in *E. coli* and *Salmonella*. Our results suggest that the *bla*_CMY-2_ plasmid lineage appears to have derived from an ancestral IncA/C plasmid type harboring *floR-tetAR-strAB* and Tn*21*-like accessory modules. Evidence is mounting that IncA/C plasmids are widespread among enteric bacteria of production animals and these emergent plasmids have flexibility in their acquisition of MDR-encoding modules, necessitating further study to understand the evolutionary mechanisms involved in their dissemination and stability in bacterial populations.

## Introduction

The use of antimicrobial agents in agriculture has been scrutinized over the past two decades because of their potential detrimental effects on animal and human health. Although the administration of antibacterial agents is an effective means to control bacterial infections, the use of antibiotics in agriculture is not limited to disease treatment and control; they are also used to prevent disease and to promote growth. Such use is postulated to facilitate the emergence of multidrug resistant bacteria isolated from animal sources (e.g., non-typhoidal *Salmonella* spp., *Escherichia coli* and other food-borne pathogens), and the dissemination of their multidrug resistance (MDR)-encoding determinants to other susceptible bacteria through horizontal gene transfer. The dissemination of MDR via conjugative plasmids can potentially limit future therapeutic options for treating infections in animals and humans [1], [2], [3], [4], [5], [6], [7].

Horizontal transfer of individual or arrays of resistance genes occurs mainly through the acquisition of conjugative plasmids, integrons, and transposons in enteric bacteria. Bacterial plasmids are self-replicating, extrachromosomal replicons, and as such they are key agents of genetic change in microbial populations. Besides conferring resistance to antibiotics, naturally occurring plasmids promote the spread of a variety of traits, including resistance to mercury and other heavy metals, virulence, fitness, and the metabolism of unusual compounds [6], [7], [8], [9], [10], [11], [12]. In recent years, there has been growing interest in the study of plasmids belonging to the IncA/C incompatibility group, mainly because of their ability to confer resistance to a diverse group of antimicrobial agents and their broad host range. IncA/C plasmids have been identified in numerous bacterial species, including *Aeromonas hydrophila* [13], [14], *Yersinia pestis* [15], [16], *Photobacterium damselae* subsp. *Piscicida* [17], [18], *Klebsiella pneumoniae* [19], *Vibrio cholera* [20], [21], *E. coli* [22], *A. salmonicida* [1], and *S. enterica* [8], [22]. Analysis of the completed sequences of these plasmids has revealed that, with the exception of accessory components containing resistance-encoding elements, they were virtually identical to one another [14], [16], [22]. Among the genes identified within the IncA/C plasmid accessory regions are those encoding for resistance to tetracycline (*tetA)*, chloramphenicol/florfenicol (*floR*), streptomycin/spectinomycin (*aadA2*), sulfonamides (*sul1* and *sul2*), and extended-spectrum β-lactamases (*bla*_CMY-2_). In addition, the recent epidemic emergence of strains containing the *bla*_NDM-1_ metallo-beta-lactamase gene, which are resistant to all antibiotic options in humans, has been associated with the IncA/C plasmid [23].

We recently completed the sequence of an IncA/C plasmid from *E. coli* isolated from a dairy cow in Illinois. This plasmid, approximately 165 kb in size, shared strong similarities with IncA/C plasmids isolated from human-source *Salmonella*, suggesting recent movements of this plasmid type among a variety of enteric populations [22]. The widespread distribution of IncA/C plasmids among *E. coli* and *Salmonella* necessitates studying their genetic repertoire and similarities with plasmids from other bacterial populations in order to fully understand their emergence and evolution in these species. Therefore, the aim of this study was to analyze genetic differences in several IncA/C plasmids from *E. coli* recovered from differing production animal sources and geographical locations using comparative plasmid sequencing and analysis.

## Results

### Sequence overview

Four plasmids were sequenced in this study, including the resequencing of pAR060302, previously isolated from a florfenicol-resistant *E. coli* commensal isolate from a US dairy cow [22]. The remaining three plasmids sequenced were from a commensal *E. coli* strain from a dairy cow in Chile (pPG010208), an avian pathogenic *E. coli* strain from a turkey in the USA with colibacillosis (p199061_160), and a porcine enterotoxigenic *E. coli* strain from a pig in the USA with post-weaning diarrhea (pUMNK88_161). All were sequenced using high-throughput Roche 454 DNA sequencing. These plasmids were isolated from farms in different geographical locations in the U.S. and Chile (Table 1). Single contiguous sequences with at least 15-fold coverage were obtained for each plasmid sequenced using draft assembly and PCR-based gap closure. BLAST analysis of the completed nucleotide sequences confirmed that they all belonged to the IncA/C incompatibility group based upon analysis of the predicted replicon. The sequence of pAR060302 was identical to the previous sequence generated via Sanger sequencing [22]. The plasmids varied in size from 135 to 165 kb, and with the exception of the accessory regions (see below), their backbone sequences were highly conserved (>99% nucleotide sequence similarity) and syntenic. Of the predicted open reading frames, approximately 40% were of unknown function. The predicted proteins with known function were primarily associated with resistance to antibiotics and heavy metals, conjugative transfer, and replication (Table S1).

**Table 1:** General characteristics of the IncA/C plasmids sequenced in this study.

Plasmid	pAR060302	pPG010208	pUMNK88_161	p199061_160
Source location	Illinois-USA	Valdivia-Chile	Minnesota-USA	USA
Source host	Cow	Cow	Pig	Turkey
Year of isolation	2002	2004	2007	1995
Resistance phenotype	FLO-TET-CHL-SUL-AMP^A^	FLO-TET-CHL-SUL	FLO-TET-CHL-SUL-AMP	FLO-TET-CHL-SUL-AMP
Size (bp)	166,530	135,803	161,081	160,573
G+C content	53.12%	51.47%	52.59%	53.10%

A FLO = florfenicol; TET = tetracycline; CHL = chloramphenicol; AMP = ampicillin; SUL = sulfisoxazole.

### *sul2*-containing accessory region

**Figure 1:**
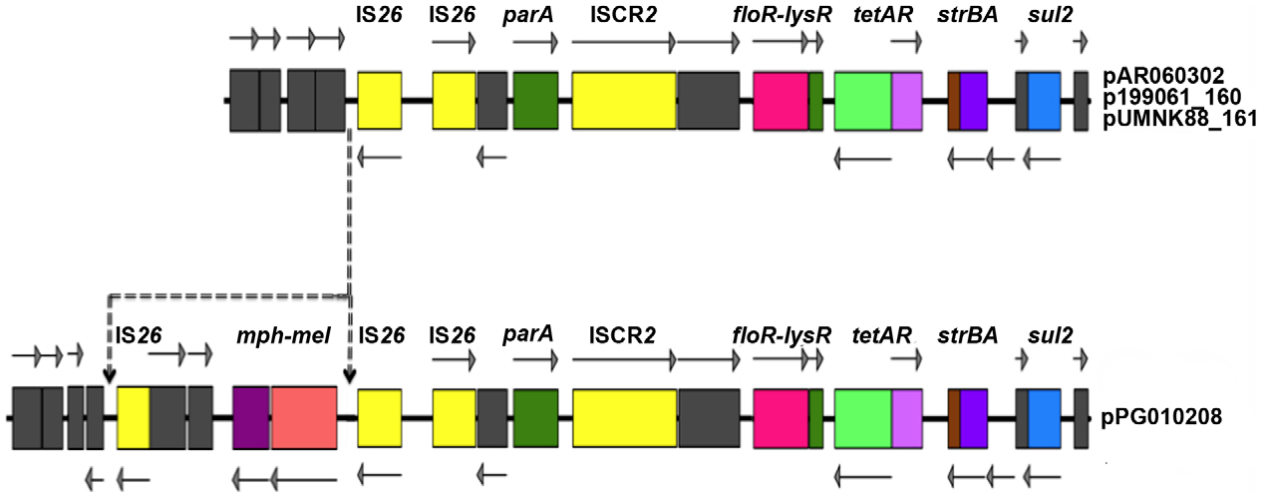
Comparison of the *sul2* regions of the plasmids sequenced in this study. Colored boxes represent predicted open reading frames as follows: grey = unknown function, yellow = mobile genetic element. The arrows indicate transcription direction. Dotted lines indicate the positions where the plasmids differ. See Table S1 for a full list of sequenced gene annotations.

As described above, the sequenced plasmids differed primarily in their accessory regions. These regions mainly included insertion sequences and transposases, class 1 integrons, antibiotic resistance determinants, and heavy metal detoxification proteins. Analysis of these regions revealed the presence of several accessory gene clusters implicated in resistance to multiple antimicrobial agents. One of these regions occurs between *repA* and a putative conjugative transfer region (designated Tra1), and is a 16-kb module containing *floR-tetA-strAB-sul2*, encoding resistance to phenicols, tetracyclines, aminoglycosides, and sulfonamides (Fig. 1). This *sul2*-containing region is identical in plasmids pAR060302, pUMNK88_161, and p199061_160, with the exception of a truncated *strB* gene in p199061_160. Also, located upstream of the *floR* gene were two copies of IS*26*, two ORFs encoding unknown functions, and an ISCR*2* element [24]. This region was also present in pPG010208; however, the *mph2* and *mel* genes encoding for macrolide resistance and an additional IS*26* element are also located upstream of this region (Fig. 1).

### Conjugative transfer and *bla*_CMY-2_-containing regions

**Figure 2:**
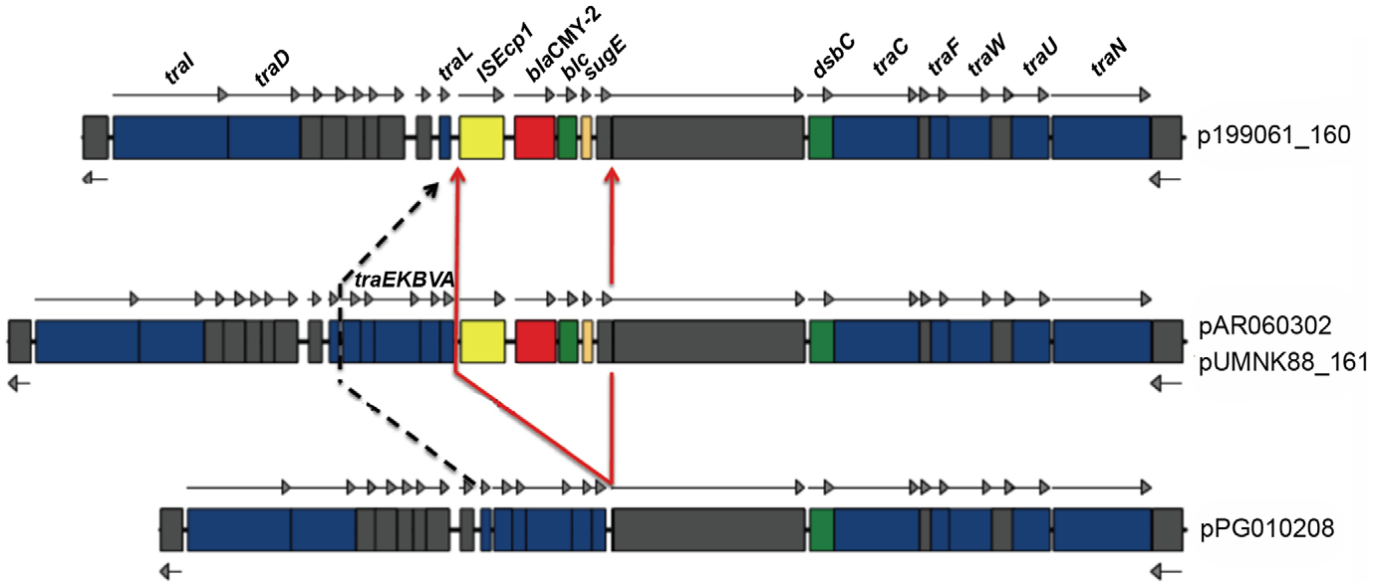
Comparison of the conjugative transfer and the *bla*_CMY-2_ containing regions of the plasmids sequenced in this study. Colored boxes represent predicted function as follows: red = antibiotic resistance, grey = unknown function, yellow = mobile genetic element, blue = transfer, green = known function. The arrows indicate transcription direction. Dotted lines and red lines indicate the positions where the plasmids differ.

Within the backbone of all of the sequenced IncA/C plasmids are two putative conjugative transfer-associated regions designated Tra1 and Tra2. The Tra1 region consists of 22 ORFs, including 9 conserved hypothetical proteins, all located in a single gene cluster (Fig. 2). In *bla*_CMY-2_-containing plasmids, one or more copies of *bla*_CMY-2_ is inserted within the Tra1 region in different locations. Among our sequenced plasmids, all except pPG010208 contained a *bla*_CMY-2_ insertion within Tra1. pAR060302 and pUMNK88_161 contained the insertion downstream of *traA*. In addition to *bla*_CMY-2_, this accessory module contains genes with homology to the *blc*, *sugE*, and *dsbC* genes as previously described [22]. Also, the insertion sequence IS*Ecp1*, belonging to the IS*1380* family, exists upstream of the *bla_CMY-2_* gene in all cases. This gene varies in size from 948 bp in the swine-source *E. coli* plasmid (pUMNK88_161) to 1,262 bp in the avian- and bovine-source *E. coli* isolates' plasmids (p199061_160 and pAR060302) (Fig. 2). In 199061_160, a region containing *traLEKBV* upstream of the insertion is absent.

### Class 1 integron-containing accessory region

A third accessory module exists in most sequenced IncA/C plasmids, separating a cluster of hypothetical genes and the Tra2 region. This region typically contains a Tn*21*-like class 1 integron structure with multiple antibiotic resistance gene cassettes and mercury resistance genes. This structure is absent from pPG010208, but present in the three other plasmids from this study inserted in identical locations. In pAR060302 and p199061_160, identical class 1 integrons are present that contain aminoglycoside resistance genes, *aadA* and *aacC*; heat-shock chaperones *groSEL*; the *qacEdelta1* and the *sul1* genes; and mercury resistance genes *merDBAPTR*. The integron region is flanked by IS*4321* elements similar to that previously described for Tn*21* [25]. In pUMNK88_161, the Tn*21*-like structure is identical to that of plasmids pAR060302 and p199061_160, except that the gene cassette region in pUMNK88_161 contains *cmlA*, encoding resistance to chloramphenicol, and *aadA2*, encoding aminoglycoside resistance.

### Transcriptional regulators

**Figure 3:**
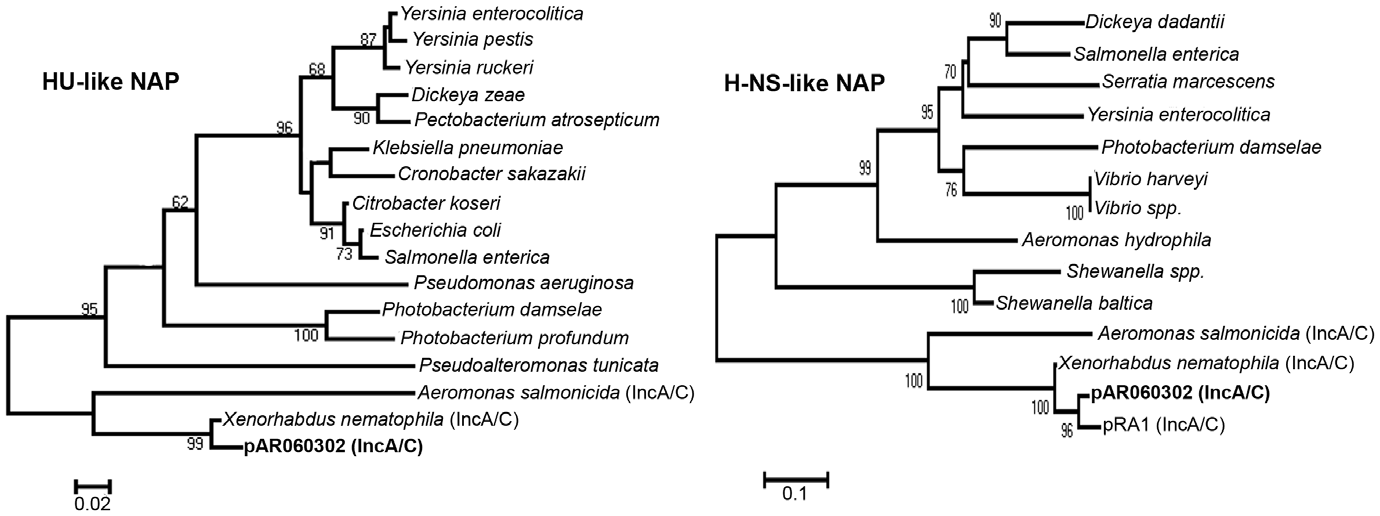
Amino acid sequence alignment of the predicted H-NS- and HU-like proteins of IncA/C plasmids. Evolutionary history was inferred using the Neighbor-Joining method. The percentage of trees in which associated taxa clustered together in the bootstrap test (500 replicates) greater than 60% are shown next to branches. Evolutionary distances were computed using the Poisson correction method. The H-NS-like protein alignment used 107 positions and the HU-like protein alignment used 90 positions. Phylogenetic analyses were conducted in MEGA4 [62].

The recent influx of available genome sequences in the public database has improved the ability to effectively annotate possible functions to predicted proteins based on inferred sequence similarity. Previously, most of the predicted proteins of the IncA/C plasmid were hypothetical proteins. Upon re-annotation of these sequences, six predicted transcriptional regulators have been identified on the IncA/C plasmid backbone. These include proteins with similarity to families HU-beta, H-NS, Xre, LysR, and LuxR. In the case of the H-NS-like and HU-like proteins, these represent novel orthologs with only 81% sequence similarity (HU-beta) and 52% sequence similarity (H-NS) to their closest matches (Fig. 3). Also, these proteins do not share any significant similarity with the previously described plasmid-encoded H-NS proteins Pmr and Sfh from IncP-7 and IncH plasmids [26], [27].

### G+C content

The G+C contents of each plasmid sequenced here were analyzed and compared to the archetypic IncA/C plasmid pRA1 isolated in 1971 from the fish pathogen *Aeromonas hydrophila* (Figure S1). Local G+C content varied from approximately 28% to 73% across the different plasmids, with two regions of high G+C content observed (∼60%). The first corresponded to the accessory module containing the *floR*, *tetA* and *sul2* genes, which is absent in pRA1. The other high G+C content fragment corresponds to the Tn*21*-like accessory regions, present on p199061_160, pUMNK88_161, and pAR060302. Additionally, two low G+C content regions were observed (∼30%) corresponding to the genes that confer resistance to macrolides and the conjugative transfer Tra1 region containing *bla*_CMY-2_. These regions of low or high G+C content were generally flanked by IS elements or inverted repeats.

### Comparison of all sequenced IncA/C plasmids

**Figure 4:**
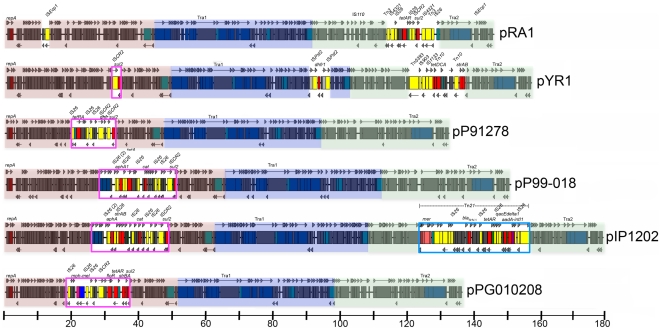
Linear maps of sequenced IncA/C plasmids lacking *bla*_CMY-2_. Core sequences are colored red (IncA/C replicon and hypothetical genes), blue (Tra1 region), and green (hypothetical genes and Tra2 region). Pink boxes depict the *sul2*-containing regions. Blue box depicts the Tn*21*-like region containing a class 1 integron. Scale is depicted in kb.

**Figure 5:**
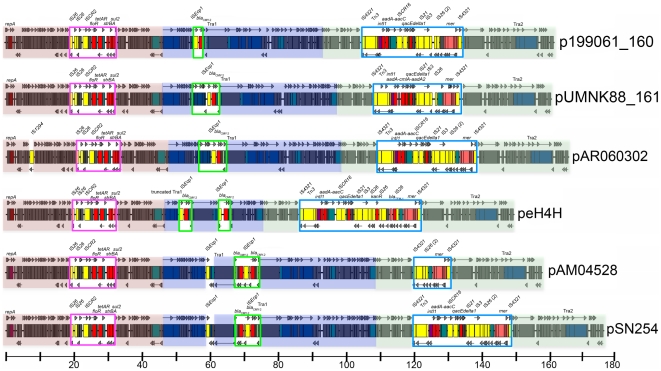
Linear maps of sequenced IncA/C plasmids possessing *bla*_CMY-2_. Core sequences are colored red (IncA/C replicon and hypothetical genes), blue (Tra1 region), and green (hypothetical genes and Tra2 region). Pink boxes depict the *sul2*-containing regions. Green boxes depict *bla*_CMY-2_ insertions. Blue boxes depict the Tn*21*-like region containing a class 1 integron. Scale is depicted in kb.

Linear maps were constructed for twelve completed IncA/C plasmids, including those from this study and from *A. hydrophila* (pRA1), *Y. ruckeri* (pYR1), *P. damselae* (pP91278 and pP99-018), *Y. pestis* (pIP1202), *E. coli* (peH4H), and *S. enterica* (pAM04528) (Figs. 4 and 5) [22]. As previously determined, the core backbones of these plasmids are all highly syntenic with no genetic rearrangements [14], [22]. The plasmids were grouped into those lacking (Fig. 4) or possessing (Fig. 5) the *bla*_CMY-2_ insertion. Eleven of twelve plasmids had *sul2* in an accessory region between *repA* and Tra1. All of the *bla*_CMY-2_ plasmids had an identical *sul2* accessory region structure containing *floR*-*tetAR*-*strBA*-*sul2*. The *sul2* regions from plasmids pPG010208, pIP1202, pP99-018, pP91278, and pYR1 all differed from the *bla*_CMY-2_ plasmids and each other. The *bla*_CMY-2_ insertions within Tra1 varied with regard to insertion location, copy number, and genetic layout. p199061_160, pUMNK88_161, and pAR060302 all contained a single *bla*_CMY-2_ insertion downstream of *traA*, with the deletion of several Tra1 genes from p199061_160. peH4H contained duplicate *bla*_CMY-2_ insertions in Tra1 with multiple truncations of the Tra1 region. pAM04528 and pSN254 both contained adjacent and inverted copies of the *bla*_CMY-2_ region downstream of *traA*.

The third accessory region site lies upstream of the Tra2 region, and generally involves integron-like elements (Figures 4 and 5). In the *bla*_CMY-2_ plasmids, all of these insertions involve intact Tn*21* or remnants thereof, inserted into identical sites. All of these regions contain a mercury resistance operon. In all *bla*_CMY-2_ plasmids except pAM04528, a class 1 integron is upstream of *mer* and varies between plasmids only with regard to gene cassette content. In pAM04528, *mer* is present but the class 1 integron appears to have been deleted. Among the non-*bla*_CMY-2_ plasmids, three (pP91278, pP99-018, and pPG010208) do not have an accessory element upstream of Tra2. One plasmid, pIP1202, contains a Tn*21*-like structure like the *bla*_CMY-2_ plasmids except that it is inverted. pRA1 and pYR1 contain insertions upstream of Tra2 that do not involve Tn*21*. In pRA1, a *sul2* element with *tetAR* is present, and in pYR1 Tn*10* and *strAB* are present.

## Discussion

It is increasingly evident that IncA/C plasmids have emerged among populations of human and animal enteric bacteria, particularly *E. coli*, *Salmonella*, and *Klebsiella* spp. To better understand the genetic structure of these plasmids in production animal *E. coli* populations, we sequenced three plasmids from *E. coli* that had been isolated from a healthy commercial dairy cow, a diseased pig, and a diseased turkey, and re-sequenced a fourth plasmid from a commercial dairy cow. These strains and plasmids were selected for their ability to confer resistance to florfenicol. DNA sequencing confirmed that all four plasmids belong to the IncA/C group. Plasmids from this group have been isolated and described previously from a variety of Proteobacteria from animals, soil, and water. These include both pathogens and commensal bacteria [8], [14], [22], [23], [28]. Despite the fact that the IncA/C plasmids sequenced thus far were isolated from different geographical locations and diverse sources, the growing collection of IncA/C plasmid sequences all share a remarkably conserved backbone with varying accessory elements collectively encoding for a broad spectrum of antimicrobial resistance. The *E. coli*- and *Salmonella*-source IncA/C plasmids sequenced thus far are quite similar in that they all have similar accessory regions, including the *sul2*-containing, *bla*_CMY-2_-containing (except pPG010208), and class 1 integron-containing modules. Temporal screening of historical *Salmonella* Newport and *E. coli* from humans and animals dating from 1940 to date suggests that IncA/C plasmids emerged after 1980 [29], [30], which is suggestive that a transfer event might have occurred prior to this time resulting in the introduction of a prototype IncA/C plasmid into these populations. However, plasmid and resistance phenotypes of IncA/C plasmids in recently isolated *E. coli* and *Salmonella* suggest that a variety of IncA/C plasmid variants exist in these populations with differing resistance phenotypes and genetic content [31], [32], [33], [34]. Therefore, it is unclear is these variants have arisen from recombinational events while in these species, or if multiple plasmid introductions have occurred.

The comparison of IncA/C plasmids from different production animals and geographic locations provides further evidence that the *bla*_CMY-2_ plasmids represent a unique IncA/C lineage that appears to be quite successful among bacterial populations, since they have been increasingly isolated and identified [35], [36], [37], [38], [39], [40], [41], [42]. Analyses of this lineage suggests that its basic structure, in addition to the IncA/C conserved components among all sequenced plasmids, also includes a *sul2* module containing *floR-tetAR-strAB* and a Tn*21*-like module. However, while the *sul2* module appears to be stably maintained among this lineage, the *bla*_CMY-2_ and Tn*21*-like regions appear to be in constant flux. The IS*Ecp1* element is associated with all copies of IncA/C-encoded *bla*_CMY-2_. IS*Ecp1*, like ISCR elements, is involved in ‘one-ended transposition’ and has the ability to mobilize itself and adjacent resistance-associated genes [43]. Mobilization and duplication of beta-lactamase genes mediated by IS*Ecp1*-like elements are well described in multiple bacterial species [44]. This helps to explain the variable duplication of the *bla*_CMY-2_-IS*Ecp1* module throughout the Tra1 region on IncA/C plasmids. The *floR* and *sul2* genes have been associated with ISCR*2* in numerous other genetic contexts [24], suggesting that ISCR*2* was involved in the introduction of the *floR-tetA-strAB-sul2* element that is conserved in this lineage of IncA/C plasmids. In the class 1 integron region, most sequenced plasmids contain an ISCR*16* element adjacent to the *groESL* genes, as previously described [24], [45], [46]. Overall, the *bla*_CMY-2_-containing IncA/C plasmids are remarkable in that they contain at least three ‘integration hotspots’ for the acquisition of accessory genetic modules; they contain multiple means of acquiring these elements, including gene cassette acquisition via integrons, classical IS-mediated acquisition via IS*26* elements; and ‘one-ended’ acquisition via IS*Ecp1* and ISCR elements; and are of apparent broad host range [47].

The backbone of the IncA/C plasmid contains a number of putative DNA binding transcriptional regulators classified as nucleoid-associated proteins (NAPs). Such proteins are named for their ability to fold chromosomal DNA and form the nucleoid within the bacterial cell, and are well studied and also known for their immense regulatory properties. NAPs are categorized into several groups, including Fis, H-NS, HU, IHF, and Lrp [48]. H-NS is known to bind to A+T-rich regions and acts as a global transcriptional repressor; HU is also a global regulator that binds to DNA non-specifically. While the most-studied NAPs are those encoded on the bacterial chromosome, a number of plasmids have also been shown to possess NAPs. The first plasmid type identified with an H-NS NAP homolog, Sfr, was the IncH plasmid R27 from *S. enterica* serovar Typhimurium [49]. The effects of this plasmid and its Δ*sfh* mutant were studied in *S*. Typhimurium. Interestingly, when pR27 was introduced into *S*. Typhimurium a limited number of chromosomal genes were differentially expressed, but the introduction of pR27Δ*sfh* resulted in a nearly 4-fold increase in the number of chromosomal genes affected [49]. Furthermore, the Δ*sfh* mutation greatly increased the fitness cost of carrying pR27 to the bacterial host. These observations were termed “stealth functions” elicited by such plasmid-encoded NAPs for their ability to silence the effects of pR27 on the host chromosome. Follow-up chromatin immunoprecipitation (ChIP) studies found that Sfh acts to bind to regions within the H-NS regulatory network and thus minimizes the effects of pR27 acquisition on the host chromosome regulatory networks [27]. A second plasmid-encoded H-NS-like protein with stealth function (Pmr) was identified on IncP-7 plasmids [26]. It is possible that the nucleoid-associated proteins encoded on IncA/C plasmids could elicit similar and immense effects on the transcriptional regulatory networks of their hosts, resulting in decreases in fitness costs and increases in host range associated with this plasmid group. However, the H-NS- and HU-like proteins identified on IncA/C plasmids are novel and only share low amino acid sequence similarity with their closest matches (Fig. 3); therefore, the roles of these proteins in such activities would need to be experimentally characterized. Certainly, further studies involving the biological mechanisms by which IncA/C plasmids succeed in various hosts are warranted, given their immense dissemination and association with pan-resistance.

A potential predecessor to the *bla*_CMY-2_ lineage of IncA/C plasmids is hinted at by the sequence of pPG010208 from a Chilean bovine-source *E. coli* isolate, which contains the *sul2* region identical to *bla*_CMY-2_ plasmids but lacks *bla*_CMY-2_ itself and lacks a Tn*21*-like accessory region. This plasmid has additionally acquired the *mel* and *mph-2* genes surrounded by two IS*26* copies upstream of ISCR*2*, present only on pPG010208 as compared to other sequenced plasmids. These genes confer resistance to macrolides, which are mainly active against Gram-positive bacteria and are considered the drug of choice for group A streptococcal and pneumococcal infections when penicillin cannot be used [50]. Similar genetic structures to this have been described on plasmid pMUR050, isolated from an *E. coli* strain from a diarrheagenic pig [51] and on the pCTX-M3, a highly conjugative plasmid responsible for the dissemination of *bla*_CTX_ genes in clinical populations of the family Enterobacteriaceae in Poland [52]. The differences observed between the Chilean isolate plasmid and other sequenced plasmids from U.S. isolates could represent an “isolation by distance” scenario, where differing local pressures could affect the acquisition of accessory elements in these plasmids. Ceftiofur is used frequently in the dairy industry of Chile, including on the farm where this isolate was obtained, but in our experience resistance to third generation cephalosporins and the *bla*_CMY-2_ gene encoding this ability are rarely identified among Chilean *E. coli* isolates. The discrepancies observed between pPG010208 and other sequenced IncA/C plasmids are not fully understood from an evolutionary and selective pressure standpoint, and deserve further study.

In addition to the accessory elements, we detected differences on the conjugal transfer system of the sequenced plasmids. In the non-*bla*_CMY-2_ plasmids, their Tra1 and Tra2 regions were generally complete and intact. However, the mosaic nature of the *bla*_CMY-2_ insertions and their duplications within the Tra1 region resulted in apparent disruptions of this region. For example, p199061_160 lacks of a 4-kb segment that includes the *traEKBVA* genes, which is present in pUMNK88_161 and pAR060302. Also, the *bla*_CMY-2_ insertion in several of these plasmids disrupts the *traA* and *traC* genes. Poole et al. studied the conjugative transferability of IncA/C plasmids containing or lacking the *bla*_CMY-2_ gene in *Salmonella*, concluding that plasmids encoding *bla*_CMY-2_ were rarely transferred compared with higher conjugation efficiencies where *bla*_CMY-2_ was absent [37]. Call et al. also reported the failure of self-conjugation for some of the IncA/C plasmids [22]. They reported that the failure of transferability of some of the IncA/C plasmids in their study was due to differences of the *tra* genes localized with *bla*_CMY-2_. Others have noted that *bla*_CMY-2_ insertions do not necessarily affect the conjugative ability of IncA/C plasmids [31]. Thus, possible transfer deficiencies conferred through *bla*_CMY-2_ acquisition, the role of co-residing plasmids in decreasing its fitness cost and increasing its conjugative frequency, and dissection of the Tra1 and Tra2 regions in conjugative transfer still need to be studied.

A key question pertaining to multidrug resistance encoded by IncA/C plasmids is their maintenance in bacterial populations in the absence of selective pressures. Third-generation cephalosporins such as ceftriaxone and ceftiofur have important applications to both human and animal health [53]. Various genes encode for proteins that confer reduced susceptibility to these antimicrobials, and *bla_CMY-2_* is commonly responsible for the resistance to these antimicrobial agents in the U.S. [22], [35], [37], [39], [54], [55]. Potential risks have been identified due to the possible co-selection of the *bla_CMY-2_* through the use of florfenicol in food animal production. Compounding this scenario is the presence of multiple means of selection, including antibiotics and heavy metals, on many IncA/C plasmids. It was recently demonstrated the long-term maintenance of IncA/C plasmids might require selective pressure, which contrasts the apparent success of this plasmid type in a variety of environmental niches including possible non-selective environments [56]. This underscores the need to elucidate the selective pressures that drive the success of this plasmid type in Enterobacteriaceae.

The presence of the *cmlA* gene on the class 1 integrons was another trait that differed between the IncA/C plasmid sequences. This gene was detected only on the swine-source *E. coli* plasmid, pUMNK88_161 (Figure 1). These results agree with the study by Bischoff et al., who found that 48 of the 90 *E. coli* isolates from swine production in Oklahoma exhibited resistance to chloramphenicol and 47 of these isolates possessed the *cmlA* gene [41]. This gene encodes a putative efflux pump that confers resistance to chloramphenicol, which has been banned in the U.S. since 1985. Thus, the presence of this gene on IncA/C plasmids is an example of an additional selection mechanisms for its widespread dissemination in commercial pig hosts and persistence in the absence of the particular selective pressures, and is aggravated by the fact that the use of any antimicrobial encoded by the IncA/C plasmid can potentially co-select for a number of additional phenotypes.

Previous studies of the IncA/C plasmids suggest that these plasmids probably did share a recent common ancestor. Fricke et al. sequenced pRA1 [14], considered the first member of the IncA/C group of MDR plasmids to be fully described [57]. This plasmid showed a reduced antimicrobial resistance spectrum, which the authors attributed to a probable minimal selective pressure. The authors proposed an evolutionary model in which each “IncA/C plasmid diverged from a common ancestor through a specific process of stepwise integration events of horizontally acquired resistance gene arrays” [14]. It appears that the *bla*_CMY-2_ plasmid lineage is such an example, where its emergence resulted from initial acquisitions of its *sul2* module, *bla*_CMY-2_ module, and Tn*21*. Further evolution of this plasmid lineage and other IncA/C lineages seems to be rapidly occurring, as recent reports have identified the New Delhi Metallo-β-Lactamase (NDM-1) occurring on or with IncA/C plasmids [23], [58]. Again, the underlying mechanisms driving the evolution and emergence of such IncA/C plasmid variants is unclear, but will likely present great challenges to human and animal health.

In conclusion, variants of broad-host-range IncA/C plasmids have emerged in a variety of bacterial species. The association of MDR with integrons, complex transposons, and ISCR elements, all on a conjugative plasmid, infers the possibility of dissemination among clinical isolates that creates opportunities for the rapid emergence of multidrug resistant bacterial clones. Strains harboring these plasmids serve as a reservoir for antibiotic resistance genes, the further spread of which could likely limit therapeutic options. Based upon the recent analyses of IncA/C plasmids revealing their genetic components and dissemination among *E. coli* and *Salmonella* of humans and production animals, future studies are essential to determine the specific mechanisms of acquisition, persistence, and dissemination of these plasmids among bacterial populations.

## Materials and Methods

### Bacterial isolates

All strains used in this study are listed in Table 1. Isolates were collected in previous studies by the investigators [9], [22]. The research in these studies complied with all relevant animal use federal guidelines and institutional policies. The strains were selected because they all harbored a large plasmid and exhibited resistance to ceftriaxone, florfenicol or tetracycline. All strains were cultured at 37°C in Tryptone soy agar (TSA) and stored in 40% glycerol at −80°C.

### Plasmid isolation and sequencing

Single colonies were inoculated into 100 mL LB broth and grown overnight at 37°C with shaking. Plasmid isolation was performed using Plasmid Midi Kit (Qiagen Inc., Valencia, CA). After purification, plasmid DNA was resuspended in sterile water and detected by electrophoresis on 0.8% agarose gels at 4°C. Ten micrograms of purified plasmid DNA was sequenced at Biomedical Genomic Center at the University of Minnesota using the Roche 454 GS-Titanium sequencing platform (454 Life Sciences, Branford, CT).

### Assembling and annotation

For each strain, sequencing reads were assembled *de novo* using SeqMan software from DNAStar (Lasergene, Madison, WI). Assembled contigs were then mapped to a reference genome (FJ621588) for arrangement of the contiguous sequences in their most likely orientation. The final gaps were closed using standard PCR. Open Reading Frames (ORFs) in the plasmids sequences were identified using GeneQuest from DNAStar (Lasergene, Madison, WI), and ORF Finder (http://www.ncbi.nlm.nih.gov/projects/gorf/), followed by complete manual inspection. Translated ORFs were then compared to known protein sequences using BLAST [59]. Those with greater than 80% homology with database protein sequences were considered matches, while hypothetical proteins with greater than 80% amino acid sequence identity to one or more previously published proteins were classified as conserved hypothetical proteins. Insertion sequences and repetitive elements were identified using IS FINDER (http://www-is.biotoul.fr/). Finished sequences are deposited in Genbank under accession numbers HQ023864 (pAR060302), HQ023861 (pPG010208), HQ023862 (pUMNK88_161), and HQ023863 (p199061_160).

### Comparative genomics

Following annotation, the assembled nucleotide sequences were analyzed to other plasmid sequences using BlastN [60]. Through this analysis, comparative linear maps (http://www.iayork.com/XPlasMap/) were created. The G+C content of each plasmid was analyzed using ARTEMIS software [61].

### Antimicrobial susceptibility testing

Wild type strains were subjected to disk diffusion to determine the susceptibilities of isolates to the following drugs: streptomycin, tetracycline, florfenicol, chloramphenicol, ampicillin, and sulfisoxazole.

## Supporting Information

Figure S1 **Sliding G+C contents of each plasmid sequenced in this study.** A = region containing *floR*, *tetA* and *sul2* genes, B = the Tn*21*-like accessory regions, C = genes that confer resistance to macrolides, and D = the conjugative transfer region together with *bla*_CMY-2_ gene.
(TIF)

Table S1 Annotated features of sequenced plasmids.
(XLS)
