# Supplementary figures and images for: Comparative Genomics of Multidrug Resistance-Encoding IncA/C Plasmids from Commensal and Pathogenic Escherichia coli from Multiple Animal Sources
Source: PLoS One. 2011 Aug 12;6(8):e23415. doi: 10.1371/journal.pone.0023415 (PMC3155540; doi:10.1371/journal.pone.0023415)

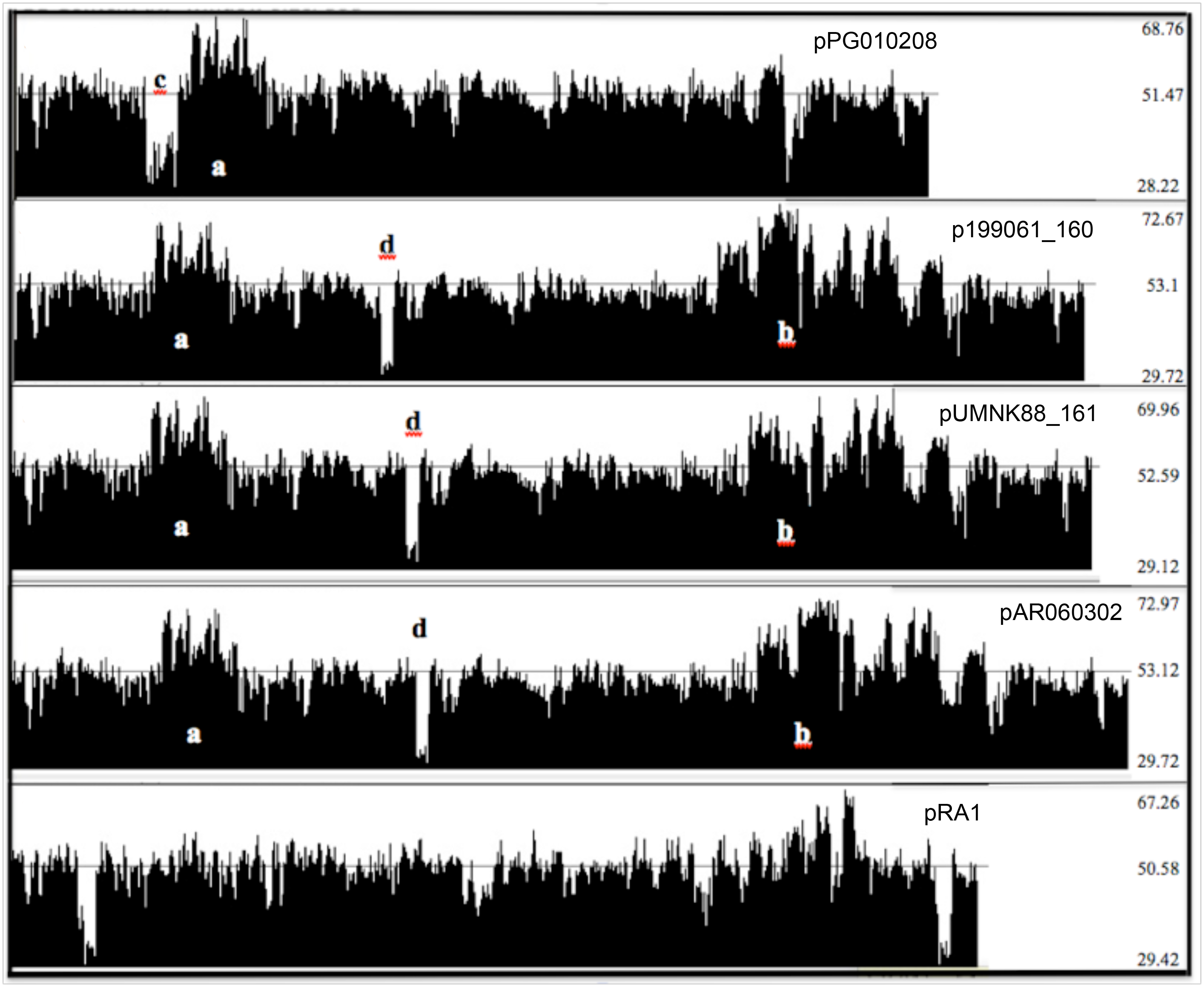

Supplement: Figure S1 — Sliding G+C contents of each plasmid sequenced in this study. A = region containing floR, tetA and sul2 genes, B = the Tn21-like accessory regions, C = genes that confer resistance to macrolides, and D = the conjugative transfer region together with bla CMY-2 gene. (TIF) [file pone.0023415.s001.tif]
